# Fast and Clean Synthesis of Nylon-6/Synthetic Saponite Nanocomposites

**DOI:** 10.3390/ma15010163

**Published:** 2021-12-27

**Authors:** Alejandro Madrid, Elena Pérez, Miguel Ángel Vicente, Vicente Rives, Raquel Trujillano

**Affiliations:** Departamento de Química Inorgánica, Universidad de Salamanca, 37008 Salamanca, Spain; ama85@usal.es (A.M.); eperez@usal.es (E.P.); mavicente@usal.es (M.Á.V.); vrives@usal.es (V.R.)

**Keywords:** nylon-6, synthetic saponite, composite, microwave irradiation, delaminated synthetic saponites

## Abstract

Nylon/saponite nanocomposites were synthesized and characterized. The nanocomposites were prepared by means of a fast, efficient, low cost, and environmentally friendly method. All of the tested preparations resulted in the pre-designed nanocomposites. To this end, delaminated saponites were directly synthesized to be used as a filler in a polymer matrix formed by nylon-6 by the in situ intercalation polymerisation of an ε-caprolactam monomer without the use of surfactants or other organic reagents to organophilise the clay, thus avoiding the drawbacks of contamination. The efficiency of the preparation method increased, and significant savings were achieved in terms of both energy reaction time, savings of 60% and 70%, respectively, by using microwave radiation as an energy source during the synthesis of the nanocomposites. In addition, given that the method that was followed avoids the use of contaminating organophilic agents, it is important to highlight the savings in reagents and the fact that there was zero environmental contamination.

## 1. Introduction

In situ intercalation polymerisation was the first method that was used to prepare nylon-6/clay nanocomposites by researchers at Toyota from the monomer ε-caprolactam [1,2,3] and previously from organophilised natural montmorillonite. The clay was swollen in a solution of the chosen monomer, which diffused into the interlayer space of the clay, and then the polymerisation process was carried out through application of heat or radiation [4].

There has been a large number of studies on the efficiency of well-exfoliated polymer-clay nanocomposites compared to conventional composites as well as on their synthesis methods, which have been discussed in several papers and literature reviews [5,6,7,8,9]. The exfoliation or delamination of clays is of particular interest because this arrangement maximizes polymer–clay interactions due to the availability of the entire surface of the layers to the polymer, leading to substantial variations in the mechanical and physical properties [10]. It has been proven that exfoliation gives rise to a complete dispersion of the nanolayers in the polymer matrix; thus, it is better than intercalation because the properties [11] of the polymer become further enhanced. This improvement is due to the coupling between the high surface area of the exfoliated clay and the polymer matrix, facilitating stress transfer to the reinforcement phase. Wu et al. [12] studied the thermal, morphological, and mechanical properties of a polyamide 6/clay nanocomposite and found that they were superior to those of carbon fiber-and glass fiber-reinforced polyamide-6 composites. This may be due to the strong interaction force that exists between the polyamide-6 matrix and the nanoclay interface. The effect of nanoscale clay on toughness is more significant than that of the fiber. Da Paz et al. [13] tested the mechanical and thermomechanical properties of polyamide 6/Brazilian organoclay nanocomposites; they found that the clay acted as a reinforcing filler and that mechanical properties such as the modulus and yield stress increase if compared to those of polyamide 6. They also proved that the heat deflection temperature values of the nanocomposites were 100% higher than those that were demonstrated by the pure polymer. On the other hand, Nayak et al. [14] showed that the reinforcement of glass fiber/epoxy composites with nanoclays improves the flexural and thermal properties of the composites. The structure and properties of polymer-nanoclay composites were described in detail by Guo et al. in a review [15] overview of polymer–nanoclay composites; in this publication, the authors reported on the synthesis routes that are commonly used to prepare these materials. In many cases, the inclusion of a highly dispersed, single-layered, delaminated clay in the polymer matrix can enhance the properties of the polymer [16]. Kakuta et al. [17] recently synthesized and functionalized organic compounds, such as pillar [5] arenes, with several cationic substituents and used them as intercalants to modify bentonite. They proved that the pillararenes exfoliate the clay by forming polyrotaxanes with poly(ethylene glycol) through host–guest interactions.

The aim of this work to synthesise and characterise nanocomposites that have been prepared by means of a fast, efficient, low cost, and environmentally friendly method. To this end, delaminated saponites were directly synthesized following the method described by Trujillano et al. [18,19,20], and they were used as a filler for a polymer matrix formed by nylon-6 by the in situ intercalation polymerisation of the ε-caprolactam monomer without the use of surfactants or other organic reagents to organophilise the clay, thus avoiding the drawbacks of contamination.

This objective seeks to increase the efficiency of clay synthesis preparation methods while avoiding the use of organic intercalants that should be removed by washing or calcination after the synthesis of the exfoliated clay, thus polluting water and/or the atmosphere. Other advantages that are sought are energy savings and decreasing the reaction time by using microwave radiation during the synthesis of the nanocomposites.

## 2. Materials and Methods

### 2.1. Materials and Preparation Procedures

NaOH, NaHCO_3_, Na_2_SiO_3_ solution (1.39 g/mL and 27% SiO_2_) MgCl_2_.6H_2_O (98–100%), AlCl_3_.6H_2_O (95–100%), and C_6_H_11_NO (ε-caprolactam 99%) were used as reagents, and all of them were obtained from Panreac; N_2_ (99.95%), O_2_ (99.995%), and He (99.995%) were obtained from L’Air Liquide Spain, S.A.

Saponites were prepared following the method proposed by Kawi and Yao [21] and that was subsequently modified by Trujillano et al. [18,19,20] with the use of microwave irradiation to accelerate the reaction.

A portion (16.8 mL, 23.45 g) of sodium silicate (Na_2_SiO_3_) aqueous solution was added to a solution (pH = 12–14) containing 10.86 g (0.27 mol) of NaOH and 19.78 g (0.23 mol) of NaHCO_3_ dissolved in 150 mL of distilled water (solution A). Another aqueous solution (B) containing 3.66 g (0.015 mol) of AlCl_3_.6H_2_O and 18.67 g (0.092 mol) of MgCl_2_.6H_2_O in 15 mL of distilled water was slowly added to solution A under continuous and vigorous stirring conditions. Once the addition was finished, the gelatinous white suspension that was obtained was magnetically stirred for one hour. All of these steps were carried out at room temperature. Then, the formed gel was sealed in a 100 mL Teflon reactor and was hydrothermally treated in a Milestone Ethos Plus microwave oven at 180 °C for 2 h while the gel was continuously stirred to avoid the platelets from stacking as much as possible. The suspension that was thus obtained was washed with distilled water until the chloride ions had been totally removed (checked by precipitation with AgNO_3_), and the suspension was then dried in an oven at 75 °C in air. This sample was labelled as SCA.

The method that was followed to prepare the clay/monomer mixture that was used as a precursor for the nanocomposites was based on the method reported by Fukushima and Inagaki [22]: a mixture comprising the synthetic saponite SCA (5%) and the ε-caprolactam monomer (95%) was prepared. To this end, 0.5 g of saponite was added to a solution of 9.5 g of ε–caprolactam in 10 mL of distilled water. This suspension was kept under constant stirring for 1 h under a nitrogen atmosphere to prevent it from becoming carbonated. The sample was dried by gentle heating in a water bath. When the sample was almost completely dry, it was moved to an oven that was set at 60 °C to ensure that it was completely dry. The sample that was obtained was called SCACapro.

Pure nylon-6 (N6) was used as a reference, and the nylon-6 nanocomposites N6S5-X (X indicates the reaction time, 6, 24 or 48 h) were obtained by heating the precursors in sealed glass ampoules in a conventional muffle furnace.

To prepare the N6 samples, 0.5 g of ε-caprolactam was placed in a glass ampoule, and two drops (approximately 0.1 mL) of distilled water were added. The ampoule was then closed and was introduced into the muffle furnace, where the polymerisation reaction was conducted upon heating at 250 °C for 6 h. 

The nanocomposites N6S5-X were obtained following the same method as the one referred to for N6, but not water was added since the water that was retained by the clay together with the protons of the surface of the layers would hopefully result in the ε-caprolactam ring opening, thus triggering polymerisation. As such, 0.5 g of SCACapro was placed in a glass ampoule with ε-caprolactam and was then placed in the muffle, where it was heated at 250 °C for 6 h to produce the polymerisation reaction. The solid that was obtained was labelled as N6S5-6. To obtain the nanocomposites N6S5-24 and N6S5-48, the same procedure was followed, the only difference being the heating time (24 or 48 h, respectively).

To reduce the working time and the amount of energy spent, the procedure that was described in the previous paragraph was repeated in order to obtain a new series of nanocomposites, which were designated as N6S5MW-X, where X represents for the reaction time (6, 8, or 10 h), by using microwave radiation to carry out the polymerisation reaction, aiming to reduce the amounts of energy and the time needed.

Table 1 summarizes the names that were given to each sample and a brief description of the preparation procedure. The samples that are fully described in this work are only those that were obtained using the minimum treatment time necessary to obtain the nanocomposite in the muffle or in the microwave oven.

### 2.2. Characterisation Techniques

Element chemical analyses were carried out by atomic absorption in a Perkin Elmer Elan 6000 ICP Mass Spectra apparatus at Servicio General de Análisis Químico Aplicado (University of Salamanca, Salamanca, Spain). The powder X-ray diffraction (PXRD) patterns were recorded in a Siemens D-500 diffractometer equipped with Diffract-AT software and a DACO-MP microprocessor, a copper (λ = 1.5405 Å) anode, and a graphite filter. The FT-IR spectra were recorded in the range of 4000–400 cm^−1^ by the KBr technique in a Perkin Elmer FT-IR-1600 spectrometer; a total of 12 scans were recorded to improve the signal-to-noise ratio at a nominal resolution of 4 cm^−1^. Specific surface area and porosity analysis were determined from the nitrogen adsorption–desorption isotherms at −196 °C and were recorded using a Gemini instrument from Micromeritics after the samples had been degassed for 2 h at 110 °C in a Flowprep 060 apparatus, which was also from Micromeritics. Differential thermal analyses (DTA) and thermogravimetric (TG) analyses were carried out simultaneously using SDT Q600 equipment from TA instrument, under a dynamic oxygen (50 mL/min) atmosphere at a heating rate of 10 °C/min.

## 3. Results

### 3.1. Synthetic Saponite

**Figure 1:**
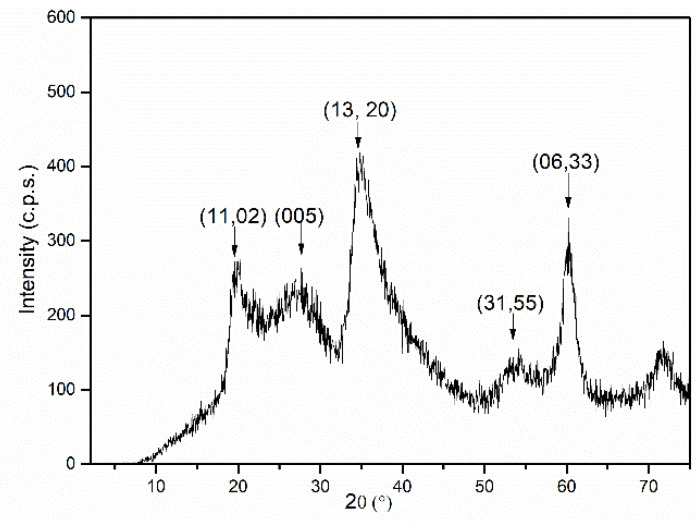
PXRD diagram of sample SCA.

The theoretical formula for saponite, referred to as half-cell, is [Si_8-x_Al_x_][Mg_6_]O_20_(OH)_4_[M^+^]x⋅nH_2_O, and in our case, considering the Si/Al/Mg ratio in the reacting mixture, the targeted formula is [Si_7_Al_1_][Mg_6_]O_20_(OH)_4_[Na_1_]⋅nH_2_O. From the chemical analysis data of the solid, and the water content that was determined from TG (vide infra), the formula [Si_6.85_Al_1.15_][Mg_6.00_]O_20_(OH)_4_[Na_1.54_]·9H_2_O·2.81Mg(OH)_2_·0.05Al(OH)_3_ was obtained. A relatively high excess of Mg was observed with respect to the available octahedral positions (6), strongly suggesting its presence as an extra-framework phase and that it had been tentatively formulated out of the formula of saponite as Mg(OH)_2_. The substitution in the tetrahedral layer is close, although slightly higher, than that of the targeted one. The value of the sum (Si + Al) is 8.05, while the layer contains 8.00 positions, so a small amount of silicon or aluminum may also be present as an extra-framework phase that was formulated out of the saponite formula as Al(OH)_3_. Thus, the prepared saponite has a high charge, about 1.15 units per half-cell, that is balanced by the interlayer Na^+^ cations and with a small excess of this element (all Na is formulated in the exchangeable positions of the clay although its excess should also be formulated out of the formula). All of the extra framework phases, which may include simple or mixed oxides that comprise the three elements that are in excess, should be amorphous, as only saponite was identified by powder X-ray diffraction (Figure 1).

A (001) diffraction peak due to saponite should be expected at around 15 Å in a well-ordered saponite, but it is not observed at all. The positions of the diffraction peaks of the sample SCA are close to those observed by Suquet et al. [23] in a Kozakov saponite (SNAT). The shifts in the positions of the diffraction peaks that are associated with basal diffractions are usually related to the nature of the exchangeable cations (concentration, size, etc.) and with the number of water molecules located between the sheets [24]. The absence of reflection (001) in the diffractogram of the SCA samples reveals that an exfoliated or delaminated clay was obtained; that is, the sheets that form the clay are totally disordered and do not present any interaction between them nor in the long-term stacking along the *z* direction. The *d* spacing value for reflection (06,33) for the SCA sample is 1.54 Å, a value that very close to that reported by Suquet et al. [23] that is and characteristic of trioctahedral smectites, which is the case for saponite [25]. This reflection originates within the layer, and it is very sensitive to the nature of the cations in octahedral positions, showing values close to 1.48 Å in the dioctahedral and close to 1.53 Å in the trioctahedral smectites, allowing the dioctahedral and trioctahedral clays to be distinguished and confirming the strongly trioctahedral character of the synthesized saponite.

**Figure 2:**
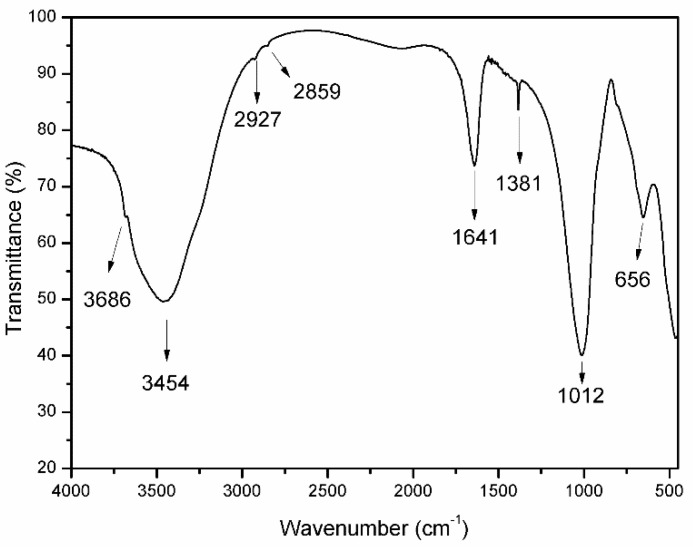
FT-IR spectrum of sample SCA.

The FT-IR spectrum of the SCA sample is included in Figure 2. The broad and intense absorption band at 3454 cm^−1^ is due to the stretching mode of the OH groups from the layers and from the H_2_O molecules; the broadness of this band is due to the hydrogen bonds between all of the responsible species (OH and H_2_O). The marked shoulder at 3686 cm^−1^ is due to the O–H stretching mode of the Mg–OH units, thus confirming the presence of a Mg-containing amorphous (as was not detected in the PXRD diagram, Figure 1) extra-framework phase, which would account for the abnormally high Mg content in the sample. The low extremely weak bands at 2927 and 2859 cm^−1^ are due to the C–H stretching modes of the organic vapours that were adsorbed as impurities from the laboratory environment.

The medium-intensity band at 1641 cm^−1^ originates from the bending of the H_2_O molecules. The intense absorption at 1012 cm^−1^ is due to the stretching mode of the Si-O units in the clay structure. In addition, a rather weak band at 656 cm^−1^ corresponds to the vibrational mode of the bonds between the oxygen and Mg^2+^ cations in octahedral positions [9,26,27]. The weak, sharp band at 1381 cm^−1^ is due to the ν_3_ stretching mode of the nitrate impurities that are associated with the KBr disc that was used to support the sample so that the spectrum could be recorded.

**Figure 3:**
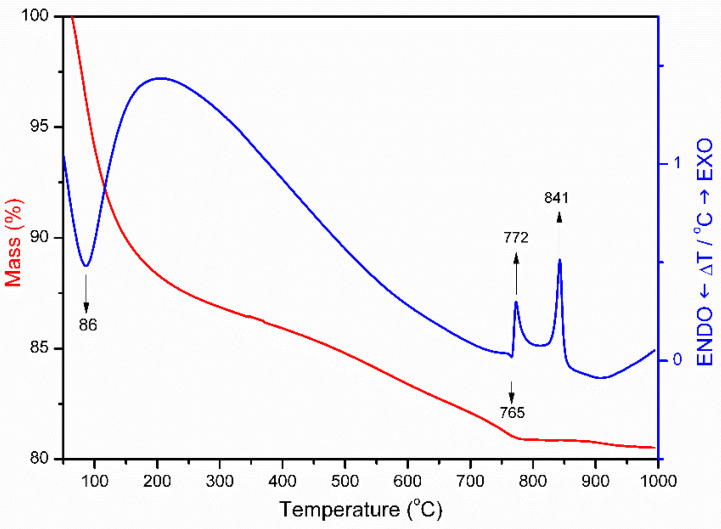
DTA–TG curves of sample SCA.

The TG curve of the SCA sample (Figure 3) shows a first mass loss of 12.5% due to the removal of the water that had been molecules adsorbed (weakly held) on the surface of the sheets that extends from room temperature up to approximately. 200 °C. Above this temperature, there is a marked difference in the slope of the curve. This process is detected in the DTA as an endothermic effect that is centred at 86 °C.

A steady mass loss amounting to approximately 5% of the initial sample mass due to removal of the hydroxyl groups from the clay structure is observed between 200 and 750 °C. At higher temperatures, between 750 and 850 °C, the DTA curve shows a very weak endothermic effect that is centred at 765 °C and that coincides with a slight (1.5%) mass loss that is immediately followed by two clearly recorded exothermic effects at 772 and 841 °C, which take place with no mass loss and that should correspond to crystallisation or to phase change processes. The first, endothermic effect at 765 °C is associated with the removal of the remaining hydroxyl groups, that is, the complete dehydroxylation of the clay layers, which leads to a recrystallization of the solid, giving rise to a new phase, a process that is associated with the first exothermic peak at 772 °C. This new phase corresponds to enstatite, a simple magnesium silicate with the formula MgSiO_3_; the second one, at 841 °C, is due to the formation of mullite (Al_6_Si_2_O_13_) [28,29].

**Figure 4:**
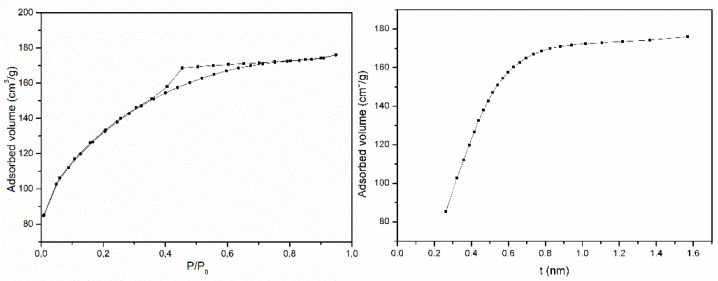
N_2_ adsorption-desorption isotherm (**left**) and *t*-plot (**right**) for sample SCA.

The textural properties of the sample were studied based on its N_2_ adsorption and desorption ability at −196 °C. The isotherm that was recorded for the SCA sample (Figure 4a) belongs to Type I of the IUPAC classification [30], a characteristic of adsorption on microporous solids. It also shows a Type H2 hysteresis loop that us usually associated with adsorption on disordered materials whose shape and pore distribution is not well defined and that indicates the existence of bottleneck-shaped pores [30].

The specific surface area, which was determined by the BET method [31], was 432 m^2^/g. The first part of the experimental data in the *t*-plot (Figure 4b) can be fitted to a straight line, and the zero intercept value can be used to determine the surface area that is equivalent to the amount of adsorption in the micropores, while the slope permits the external surface area to be determined. In this case, the values were 70 and 360 m^2^/g, respectively [30]. The curvature between both parts that are clearly defining the different sections of the curve indicates a wide distribution of the micropores [30] and the presence of mesopores. By applying the BJH method [32], the average pore diameter that was determined was 2.6 nm, and the total micropore volume that was calculated was 0.045 cm^3^/g. We can conclude that synthetic saponite is mostly mesoporous, although it also contains an appreciable volume of micropores.

### 3.2. Samples N6S5-X

**Figure 5:**
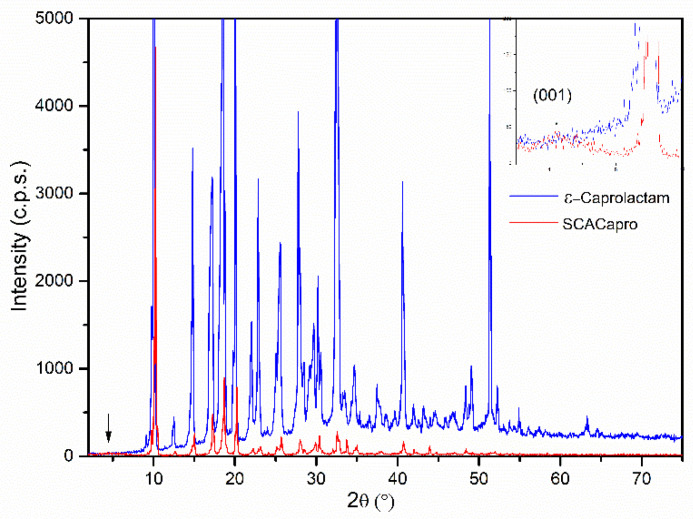
PXRD diagrams of commercial ε–caprolactam and of sample SCACapro.

The powder X-ray diffraction (PXRD) diagrams of the starting sample (SCACapro) and of commercial ε-caprolactam are included in Figure 5.

Both diagrams are very similar concerning the positions of the peaks that were recorded; however, their relative intensities differ very much from one sample to the other. A very weak peak is recorded in the PXRD diagram of sample SCACapro at 4.35° (2θ), corresponding to a spacing of 20.30 Å. No diffraction signal was recorded in or close to this position in the PXRD diagram of the SCA sample, Figure 1.

Upon the addition of molecular sized ε-caprolactam, around 7 Å, [33] to the spacing (15.32 Å) of the (001) diffraction for a reference Kozakov saponite, the value obtained, 20.3 Å, coincided with the position of the peak recorded here, which could then be tentatively ascribed to the (001) diffraction of saponite with the intercalated ε-caprolactam molecules [23,33]. This result suggests that some of the ε-caprolactam molecules were intercalated between the saponite sheets, giving rise to a partial stacking of the layers along the c-axis simultaneously. Nevertheless, the low intensity of this peak reveals a high degree of dispersion in the sheets in the monomer matrix. The positions of the other diffraction peaks coincide with those of the starting ε-caprolactam.

**Figure 6:**
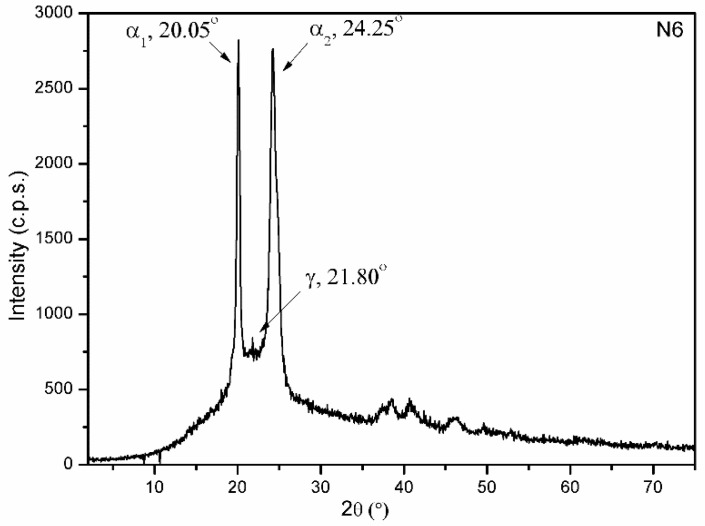
PXRD diagram of sample N6.

As mentioned above, in order to obtain a reference polymer, ε-caprolactam was submitted to the same treatment (250 °C, 6 h) that was applied to prepare the nanocomposite samples; the sample that was thus obtained was called N6. The PXRD diagram of this polymer (Figure 6) shows the characteristic diffraction peaks of nylon-6 that are described in the literature [34].

**Figure 7:**
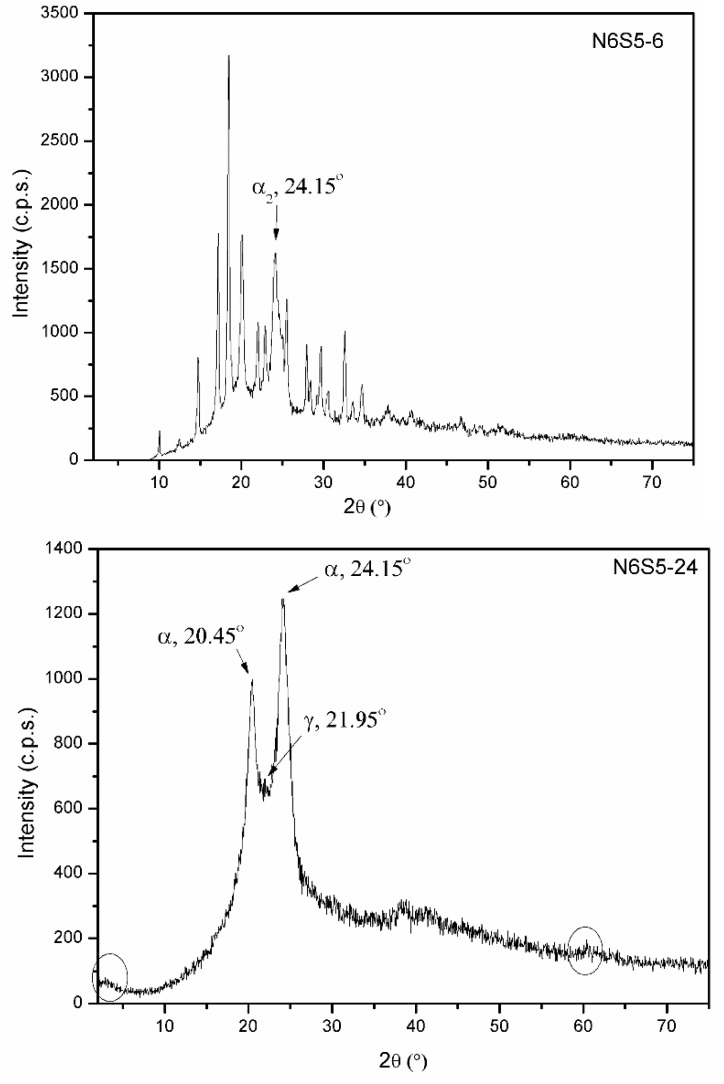
PXRD diagrams of samples N6S5–6 (**top**) and N6S5–24 (**bottom**).

Figure 7 shows the PXRD diagrams of samples N6S5-6 and NS65-24, which were obtained after the calcination of the sample SCACapro at 250 °C for 6 or 24 h, respectively, i.e., the same treatment that was used to prepare nylon-6 (here sample N6) was followed.

Most of the maxima that were recorded in the PXRD diagram of sample N6S5-6 correspond to diffraction by ε-caprolactam, and, in addition, the somewhat broader peak at 24.15° (2θ) corresponds to the second diffraction peak of the crystalline phase α of nylon-6 (α_2_) [34]. The first diffraction peak of the α phase of nylon (α_1_) is expected at 2θ = 20.05°, but its results are obscured by the much stronger diffraction peak of the monomer.

The fact that the diffraction maxima corresponding to free ε-caprolactam molecules are still recorded in the PXRD diagram of sample N6S5-6 indicates that calcination for 6 h at 250 °C is not enough to produce the complete polymerisation of ε-caprolactam. To complete the reaction, the temperature or the reaction time could be increased, and we opted to increase the reaction time. A new sample was prepared following the same process, but in this case, the reaction time was extended up to 48 h, as described in previous studies [35]. The PXRD diagram that was recorded for this sample (not shown) almost exclusively shows two sharp diffraction maxima at 20.24 and 24.05° (2θ), which correspond to the diffractions α_1_ and α_2_, respectively, of the α-phase of nylon-6. Moreover, the complete absence of any diffraction maxima due to the ε-caprolactam monomer confirmed that 48 h is a long enough reaction time to achieve the complete polymerisation of ε-caprolactam in order to produce a polymeric matrix.

**Figure 8:**
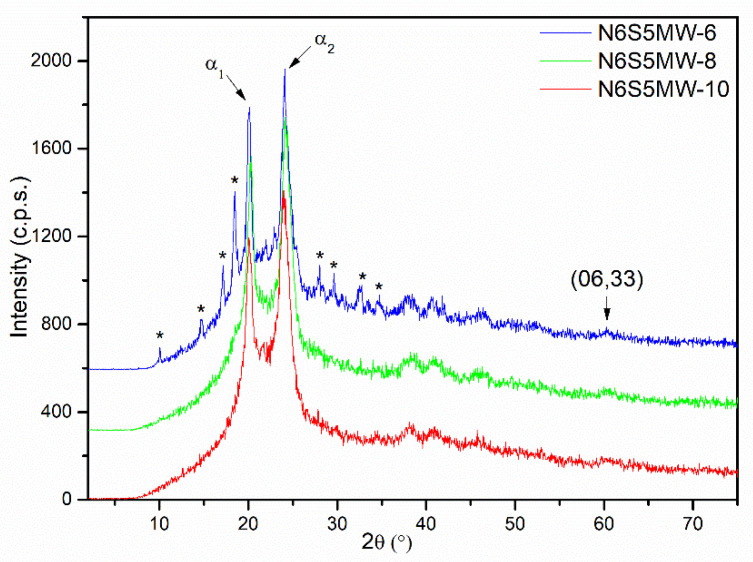
PXRD diagrams of samples N6S5MW-6, N6S5MW-8, and N6S5MW-10 (* peaks due to ε-caprolactam).

Once we checked that complete polymerisation had not occurred at 6 h but that it could be achieved after a 48 h reaction, we tried to optimize the procedure by using an intermediate reaction time, specifically 24 h, thus obtaining sample N6S5-24. The PXRD diagram of this sample (Figure 8) is identical to that of sample N6S5-48 and clearly shows the diffraction peaks of the α-phase of nylon (α_1_ and α_2_) at of 20.45° and 24.05° (2θ), respectively, in addition to a weak diffraction peak at 21.80° (2θ) due to the γ phase of the polymer [34].

It is worthwhile to mention the complete absence of any diffraction peak of the ε-caprolactam monomer, thus concluding that heating for 24 h is enough for its complete polymerisation. Other experiments (not shown here) have confirmed that shorter reaction times (between 6 and 24 h) lead to incomplete polymerisation.

The PXRD diagram for sample N6S5-24 in Figure 7 also shows two extremely weak diffraction maxima at 3.15 and 60.35° (2θ) due to diffraction by saponite planes (001) and (06,33), respectively; the corresponding d-spacings are 28.04 and 1.53 Å, respectively. The position of this second peak confirms the presence of saponite layers in the composite. The value for the first peak is much larger than the value reported [23] for Kozakov saponite (15.32 Å) and that was found for the sample SCACapro (20.30 Å), where the ε-caprolactam molecules were intercalated; this increase suggests that nylon formed in the saponite interlayer upon the polymerisation of the intercalated ε-caprolactam monomers without previous organophilisation of the clay, thus providing a method that is new, cleaner, faster, and cheaper than the conventional one that was formerly used to prepare this sort of nanocomposite [1]. Moreover, the extremely low intensity of the peak due to diffraction by the plane (001) confirms a high degree of dispersion in the clay particles in the polymer matrix.

### 3.3. Samples N6S5MW-X

In order to accelerate the reaction and to minimize the use of energy, several tests were carried out using microwave radiation (MW) instead of conventional heating in an oven as a heat source. The SCACapro precursor was placed in a glass ampoule, closed, and submitted to microwave treatment at 250 °C for 6, 8, and 10 h to obtain the samples N6S5MW-6, N6S5MW-8, and N6S5MW-10, respectively. As with the previous set of samples, a short (6 h) reaction time was used first and then a long (10 h) reaction time, and once we determined that complete polymerisation had been achieved, we tested for intermediate reaction times (8 h). The PXRD diagrams of the three samples are included in Figure 8.

The diagram for sample N6S5MW-6 shows the α_1_ and α_2_ diffraction peaks corresponding to the α-phase of nylon-6 at 20.15° and 24.10° (2θ), respectively, in addition to rather weak diffraction peaks that are characteristic of the crystalline phase of ε-caprolactam. As with the previous set of samples, which were prepared by conventional heating, the 6 h reaction was not long enough to produce complete ε-caprolactam polymerisation, i.e., the polymerisation reaction took place but not completely.

In a second step, the glass ampoule was submitted to microwave irradiation at 250 °C for 10 h, leading to sample N6S5MW-10. In this case, which can be seen Figure 8, no diffraction maxima due to monomeric ε-caprolactam were recorded, and the only diffraction maxima shown in the diagram correspond to the α and γ phases of nylon (the peak corresponding to the second phase was extremely weak) and to the clay. That is, 10 h treatment at 250 °C was enough for complete polymerisation to occur.

Tests using shorter reaction times showed that 8 h (sample N6S5MW-8, Figure 8) is the minimum time required for complete polymerisation at 250 °C.

We can then conclude that polymerisation at 250 °C under microwave irradiation merely requires one third of the time that is usually needed when using conventional heating to prepare saponite–nylon nanocomposites with well-dispersed and non- or poorly stacked saponite layers.

**Figure 9:**
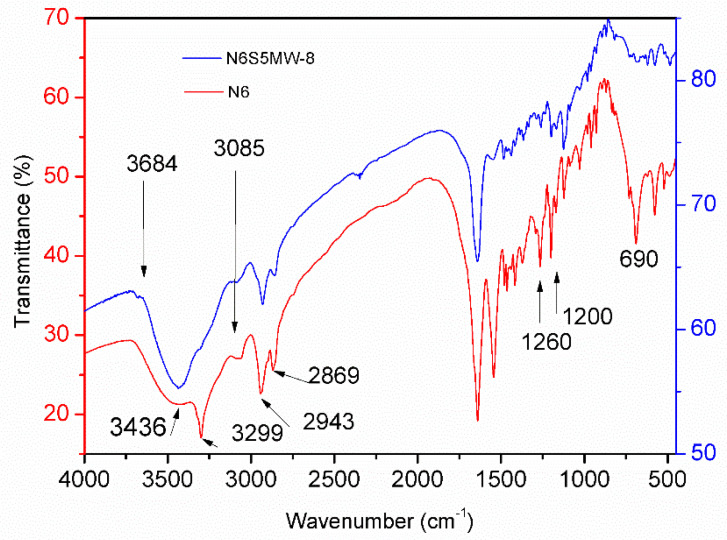
FT-IR spectra of samples N6 and N6S5MW-8.

The FT-IR spectra of sample N6S5MW–8 and of sample N6 (used as a reference) are included in Figure 9; both spectra show a series of common bands that are characteristic of the nylon-6 polymer. The positions of the bands and their ascriptions are included in Table 2.

An extremely weak band at 3684 cm^−1^, seen is Figure 9, is due to the stretching mode of the free (no hydrogen bonded) OH groups that are bonded to metal cations from the clay sheets. This band is only recorded in the FT-IR spectrum of the nanocomposite, confirming the presence of the clay sheets in the solid. A broad band at 3436 cm^−1^ in the spectrum of sample N6S5MW-8 is due to the combination of the free N–H vibrational mode and the vibrational mode of the O–H bond in the water molecules. This band is recorded in the spectra of both samples: N6 and N6S5MW-8. A shoulder due to the N–H bonds that are involved in the formation of the hydrogen bonds [36] is recorded at 3299 cm^−1^ in the spectrum of sample N6S5MW-8; however, in the spectrum of reference sample N6, this shoulder is observed as a well-defined band at 3299 cm^−1^, confirming the existence of structural differences between both solids due to the introduction of the clay sheets in the polymer matrix. The band that is recorded at 3085 and 3088 cm^−1^ for samples N6 and N6S5MW-8, respectively, is due to an overtone in the amide group band.

The bands that are recorded at 2943 and 2869 cm^−1^ in the FT-IR spectrum of sample N6 are due to the antisymmetric and symmetric vibration modes of the C–H units in the methylene groups, respectively [37]. These bands are observed at 2930 and 2858 cm^−1^ in the FT-IR spectrum of sample N6S5MW-8; the shift to lower wave numbers may be due to conformational changes in the lateral interactions between the polymer chains as a consequence of the presence of the clay sheets [38]. Two bands are recorded close to 1628 and 1547 cm^−1^, the first one due to the C=O stretching mode of the carbonyl groups and the second one to the N–C stretching mode. Two bands that can be ascribed to the amide group vibrations in the α-phase of nylon-6 (amide III bands) are recorded at around 1260 and 1200 cm^−1^ in both spectra, together with a series of bands at 1120, 1129, and 960 cm^−1^, due to the stretching and bending vibrations of the structural methylene groups. The band at 928 cm^−1^ is due to the C–C bond vibrations between the carbonyl carbon atom of the amide group and the carbon atom of the methylene group that are bound to the amide groups.

In the lower wavenumber range, a band at 730 cm^−1^ is observed in both spectra and corresponds to the rocking vibration of the methylene groups of the polymer chains. The band at 690 cm^−1^ is due to the vibration of the N–H bond (amide V). Finally, the bands that are recorded at 620 and 576 cm^−1^ are due to the vibrations of the carbonyl group in the γ and α phase of nylon-6, respectively. The band that is attributed to the γ phase is more intense in the spectrum of sample N6S5MW-8 than it is in the spectrum of sample N6, whereas the opposite occurs for the band that can be attributed to the α phase; this suggests that the introduction of a small amount of clay layers dispersed in the polymer structure favours the formation of the γ-phase of nylon-6 versus the predominant α-phase in pure nylon-6 [39].

**Figure 10:**
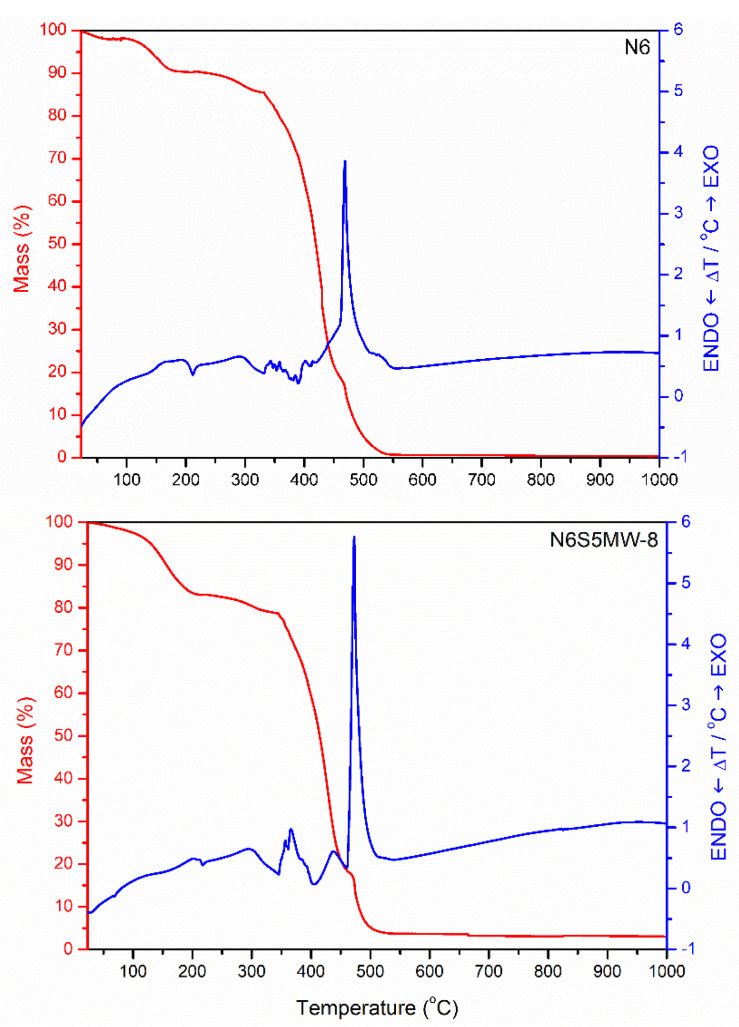
TG–DTA curves of sample N6 (**top**) and N6S5MW-8 (**bottom**).

The TG and DTA curves for samples N6S5MW-8 and N6 (for comparison) are included in Figure 10. A mass loss of close to 5% of the initial mass sample is observed between 25 and 115 °C due to the removal of physisorbed water molecules; this loss is associated with a weak endothermic effect in the DTA curve that is centred at 70 °C. After this mass loss, a further loss that is close to 10% occurs due to the removal of the water occluded between the layers.

A sudden mass loss starts at around 350 °C and extends up to 520 °C, corresponding to approximately 70% of the initial sample mass; this loss is due to combustion of the polymer, as confirmed by the very strong exothermic effect that is recorded in the DTA curve at around 475 °C. 

These thermal effects are also recorded in the curve for sample N6, but these are shifted towards somewhat lower temperatures, indicating that the polymer is thermally more stable when forming the nanocomposite than by itself. [40]. No thermal effect nor mass loss is recorded above 520 °C. The residual mass amounts to only approximately 3% of the initial mass loss and corresponds to the dehydrated and dehydroxylated clay. Of course, the final mass of N6 is zero.

**Figure 11:**
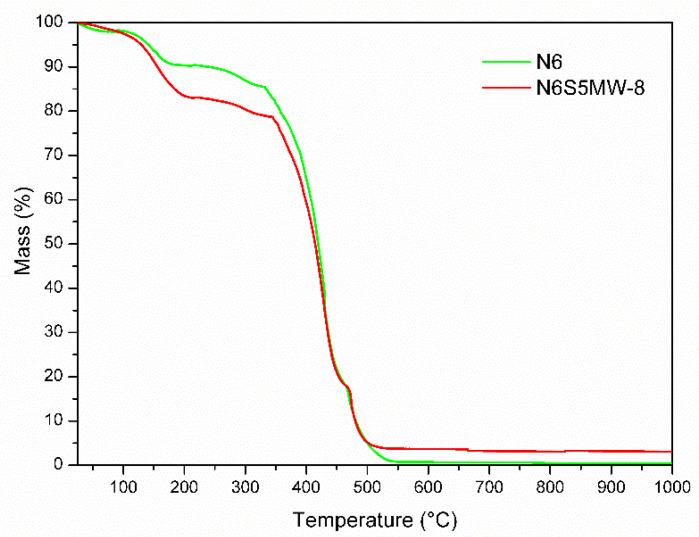
TG curves of samples N6 and N6S5MW-8.

Finally, Figure 11 includes the TG curves of both solids, allowing for an easy comparison between them. As it can be seen, several differences are observed when comparing the TG curves for these samples. First, the mass loss between 25 and 170 °C for sample N6 is 10%, whereas for N6S5MW-8, it is between 15 and 20% and takes place between 25 and 199 °C, from which it can be concluded that the nanocomposite has a larger water content due to the water molecules that are located between the saponite sheets.

Another noticeable difference that indicates the improvement in the thermal properties of the polymer thanks to the presence of these clay layers is that the decomposition of pure nylon-6 stars at 160 °C, whereas in the nanocomposite, it begins at 199 °C. This onset of decomposition is marked by the same endothermic effect in the DTA curves (Figure 10) of both solids. The improvement in thermal stability of the polymer is also observed from the beginning of the combustion of both solids, which takes place at 325 °C for N6 and at 344 °C for the nanocomposite, which is associated with their respective endothermic effects in both cases.

## 4. Conclusions

Exfoliated synthetic saponites were obtained by a hydrothermal method under microwave irradiation. The ε-caprolactam monomer was intercalated in the interlayer space of saponite in an aqueous suspension without the use of any organic compound to decrease the hydrophilicity of the clay surface. The polymerisation of the lactam in the nanocomposite (5% *w/w*) was achieved by heating the samples in a furnace in the absence of water, with the reaction being initiated by the water molecules and hydroxyl groups of the clay surface. Complete polymerisation to nylon-6 was achieved after 24 h at 250 °C, leading to swelling of the saponite layers and the almost complete delamination of the saponite layers in the organic matrix. However, heating under microwave irradiation at 250 °C for 8 h was enough to achieve a complete polymerisation as well as total the disordering of the saponite layers in the organic matrix of the nanocomposite. The use of microwave irradiation strongly decreases the reaction time required for this process (8 h) when compared to conventional hydrothermal treatment (24 h). It should be also noted that in both cases, no water was needed to trigger the reaction, which was initiated by the water molecules and the hydroxyl groups of the saponite surface.

## Figures and Tables

**Table 1:** Preparation procedure and naming of the samples studied.

Sample	Description	Procedure
SCA	Delaminated synthetic saponite	MW + stirring
SCACapro	SCA intercalated with ε-caprolactam	Aqueous suspension
N6	Nylon-6	Polymerisation of ε-caprolactam in muffle/6 h
N6S5-6	Nylon-6/SCA (5% *w/w*) nanocomposite	Polymerisation of SCACapro in muffle/6 h
N6S5-24	Nylon-6/SCA (5% *w/w*) nanocomposite	Polymerisation of SCACapro in muffle/24 h
N6S5-48	Nylon-6/SCA (5% *w/w*) nanocomposite	Polymerisation of SCACapro in muffle/48 h
N6S5MW-6	Nylon-6/SCA (5% *w/w*) nanocomposite	Polymerisation of SCACapro in MW oven/6 h
N6S5MW-8	Nylon-6/SCA (5% *w/w*) nanocomposite	Polymerisation of SCACapro in MW oven/8 h
N6S5MW-10	Nylon-6/SCA (5% *w/w*) nanocomposite	Polymerisation of SCACapro in MW oven/10 h

**Table 2:** FT-IR bands positions (cm^−1^) and ascriptions of N6 and N6S5MW-8.

N6	N6S5MW-8	Vibrational Mode
3684	3684	ν_O–H_ (free)
3436	3436	ν_N–H_ + ν_O–H__(water molecules)_
3299	3299	ν_N–H_
3085	3088	ν_N–H__(amide group overtone)_
2943	2930	ν_C–H-__ass_
2869	2858	ν_C–H-__sym_
1628	1628	ν_C=O_
1547	1547	ν_N–C_
1260, 1200, 1120, 960	1260, 1200, 1120, 960	ν_C–H_ and δ_C–H_
928	928	ν_C–C_
730	730	ν_roc–__C–H_
690	690	ν_N–H__(amide V)_
620	620	ν_C=O (γ)_
576	576	ν_C=O (α)_
